# Crystal structures of fisetin dihydrate and luteolin monohydrate: crystallization from ethanol–water mixtures

**DOI:** 10.1107/S2056989026006353

**Published:** 2026-06-26

**Authors:** Neta Caspin, Natalia Fridman, Avi Shpigelman

**Affiliations:** a Schulich Faculty of Chemistry, Technion - Israel Institute of Technology, Haifa 3200003, Israel; b Faculty of Biotechnology and Food Engineering, Technion - Israel Institute of Technology, Haifa 3200003, Israel; Katholieke Universiteit Leuven, Belgium

**Keywords:** fisetin dihydrate, luteolin monohydrate, flavonoid hydrates, aglycon flavonoids, single-crystal X-ray diffraction

## Abstract

The crystal structures of two hydrates of aglycon flavonoids, fisetin dihydrate [systematic name: 2-(3,4-di­hydroxy­phen­yl)-3,7-dihy­droxy-4*H*-1-benzo­pyran-4-one dihydrate, C_15_H_10_O_6_·2H_2_O, *P*2_1_, *Z* = 2] and luteolin monohydrate [systematic name: 2-(3,4-di­hydroxy­phen­yl)-3,7-dihy­droxy-4*H*-1-benzo­pyran-4-one monohydrate, C_15_H_10_O_6_·H_2_O, *P*4_1_2_1_2, *Z* = 8] were determined by single-crystal X-ray diffraction (SCXRD) and are reported for the first time. The two crystal forms were obtained from ethanol–water mixtures. These structures provide a foundation for future studies on the thermodynamic role of water mol­ecules in crystal stability and packing, enabling further investigation of hydration effects on flavonoid solubility and stability.

## Abstract 2

The crystal structures of fisetin dihydrate and luteolin monohydrate, obtained from ethanol-water mixtures, were determined by single-crystal X-ray diffraction.

## 1. Chemical context

Flavonoids are a subgroup of polyphenolic compounds and are considered significant contributors to the health benefits of plant-based foods (Šamec *et al.*, 2021 ▸; Panche *et al.*, 2016 ▸). Recent reviews have emphasized that understanding the crystal structures of flavonoids is essential for predicting their surface properties and inter­molecular inter­actions, which directly influence solubility, stability and crystal morphology (Xu *et al.*, 2023 ▸). Furthermore, co-crystal studies demonstrate the structural diversity of flavonoids and highlight the need for systematic crystallographic investigations to optimize their functional performance in food and pharmaceutical applications (He *et al.*, 2016 ▸). An example of this is the work done by Klitou and co-workers (Klitou *et al.*, 2019 ▸, 2020 ▸, 2022 ▸, 2023 ▸) that has highlighted the link between the crystal structure of quercetin (an aglycon flavonoid) and its crystallization behavior, including synthonic models that explain how mol­ecular information influences crystal packing and impacts crystallization processes. Similarly, several crystal structures containing fisetin have been reported, including fisetin (CCDC 1884089; Chadha *et al.*, 2019 ▸) and co-crystals with caffeine (CCDC 986281; Sowa *et al.*, 2014 ▸), nicotinamide (CCDC 986280; Sowa *et al.*, 2014 ▸), glutaric acid (CCDC 1884086), malic acid (CCDC 1884087), and theophylline (CCDC 1884088; Cox *et al.*, 2003 ▸). Luteolin has been shown to form a hemihydrate structure (CCDC 217463; Chadha *et al.*, 2019 ▸) and co-crystals with l/d-proline (CCDC 1444362 and 1446362; He *et al.*, 2016 ▸) and with 4,4′-bi­pyridine and ethyl acetate (CCDC 2385531; Xu *et al.*, 2025 ▸). In addition, there is evidence that luteolin can form co-crystals with isoniazid and caffeine (Luo *et al.*, 2019 ▸). These examples illustrate the ongoing inter­est and research in flavonoid solid forms, while highlighting that much remains to be discovered about their polymorphic and co-crystal landscapes. Comprehending the solid structures of flavonoids is crucial for controlling their functional properties in food and pharmaceuticals, providing a foundation for further thermodynamic and kinetic investigations.
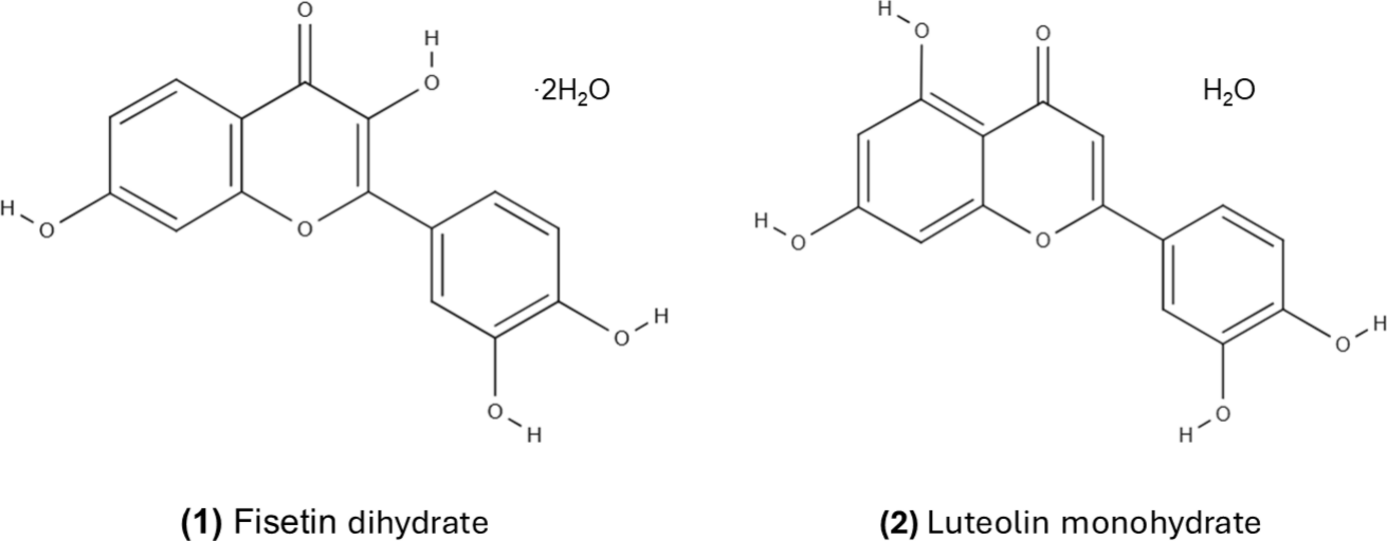


## 2. Structural commentary

**Figure 1:**
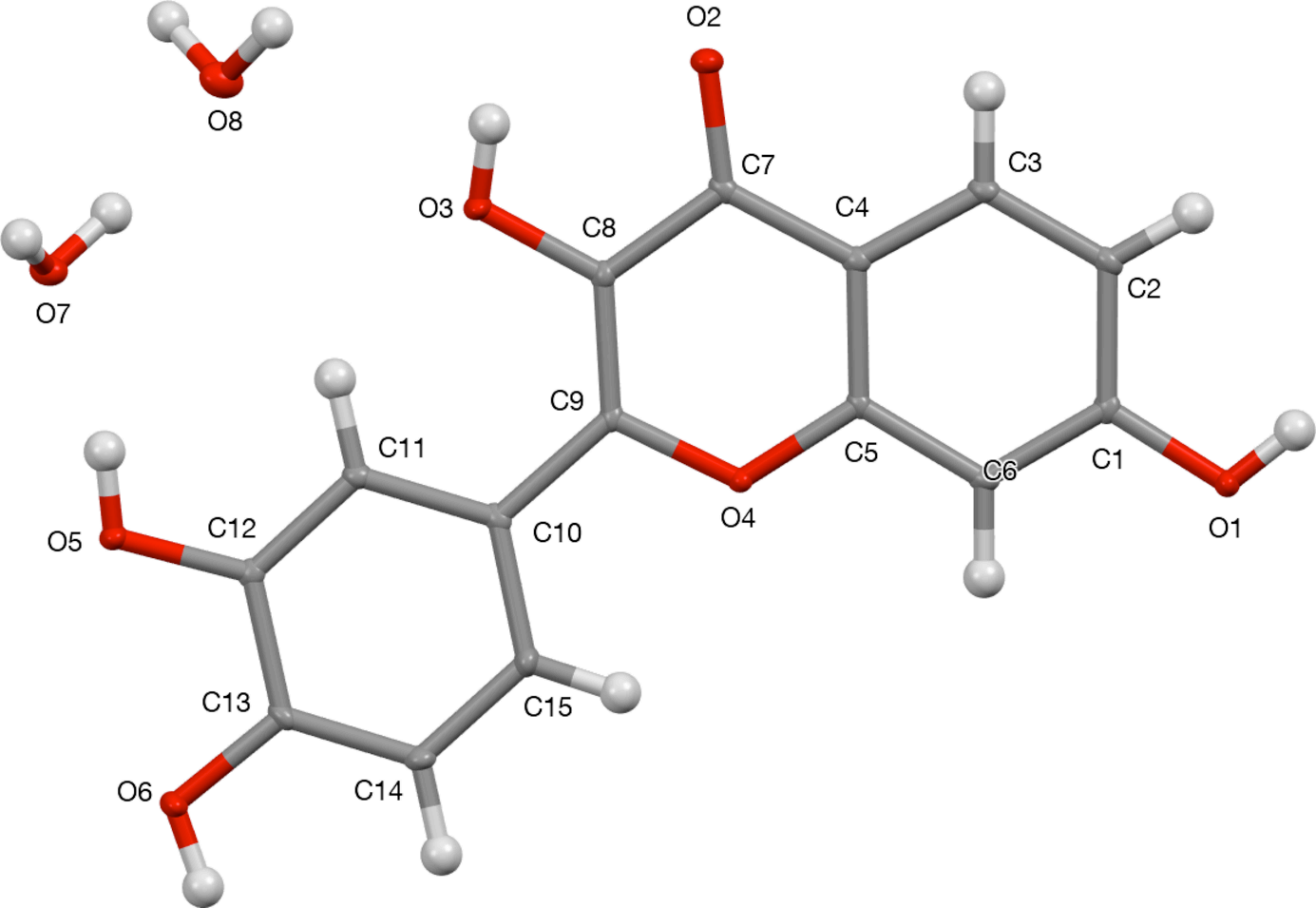
Mol­ecular structure of fisetin dihydrate (**1**), showing displacement ellipsoids at the 50% probability level.

Fisetin dihydrate (**1**) crystallizes in the monoclinic space group *P*2_1_ with one fisetin mol­ecule and two water mol­ecules in the asymmetric unit (Fig. 1 ▸). The dihedral angle between the C1–C9/O4 fused ring system and the C10–C15 ring 4.76 (10)°, indicating an almost planar conformation. An intra­molecular O3—H3⋯O2 hydrogen bond (Table 1 ▸) is observed, forming an *S*(5) ring motif. The relatively small O—H⋯O angle (114°) reflects the geometric constraints imposed by the five-membered ring.

**Figure 2:**
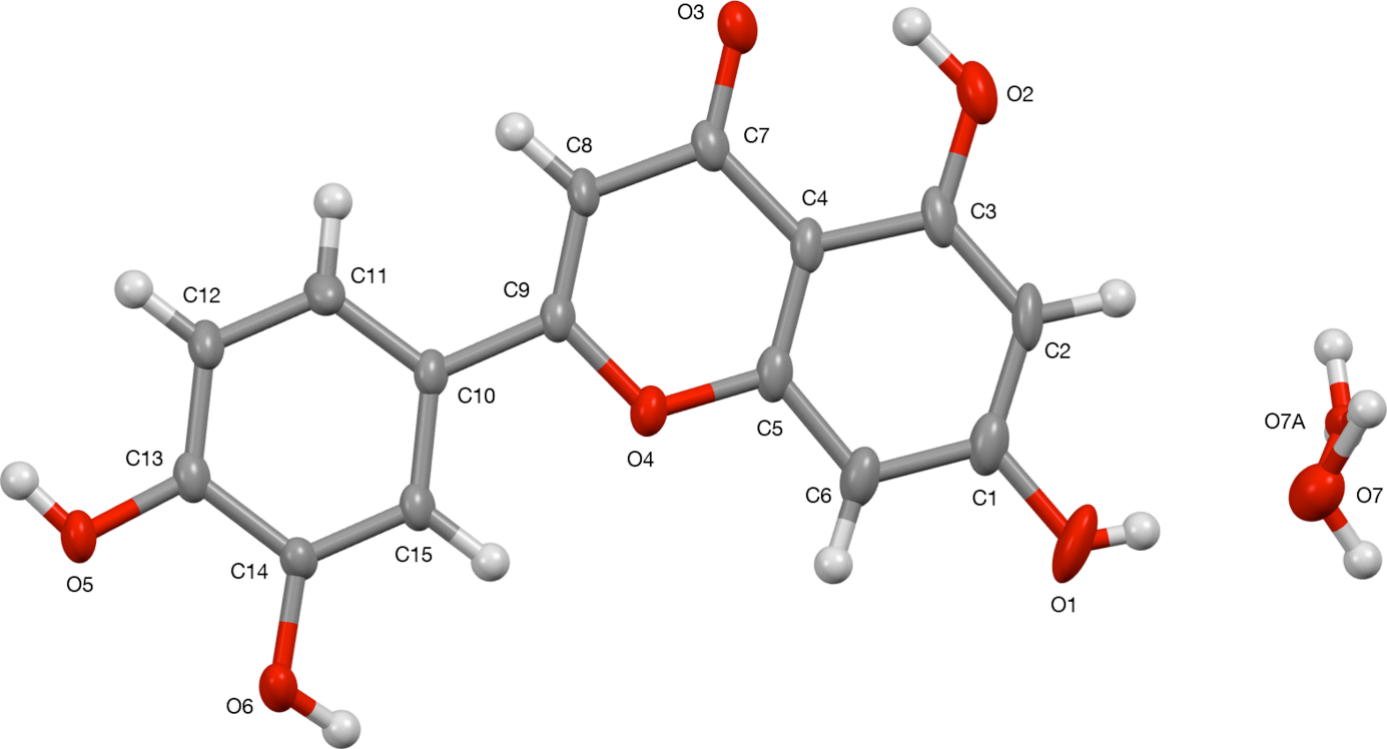
Mol­ecular structure of luteolin monohydrate (**2**), showing displacement ellipsoids at the 50% probability level.

Luteolin monohydrate (**2**) adopts the tetra­gonal space group *P*4_1_2_1_2, containing one luteolin mol­ecule and one water mol­ecule per asymmetric unit (Fig. 2 ▸). The water mol­ecule in (**2**) is disordered over two positions [occupancies 0.68 (4) and 0.32 (4)]. An intra­molecular O3—H3⋯O2 hydrogen bond is observed (H⋯O = 1.83 Å; Table 2 ▸), forming an *S*(6) ring motif, which consolidates the mol­ecular conformation. The dihedral angle between the rings is 1.18 (14)°, showing that the mol­ecule is essentially planar. Bond lengths and angles are within expected ranges for flavonoids.

## 3. Supra­molecular features

**Figure 3:**
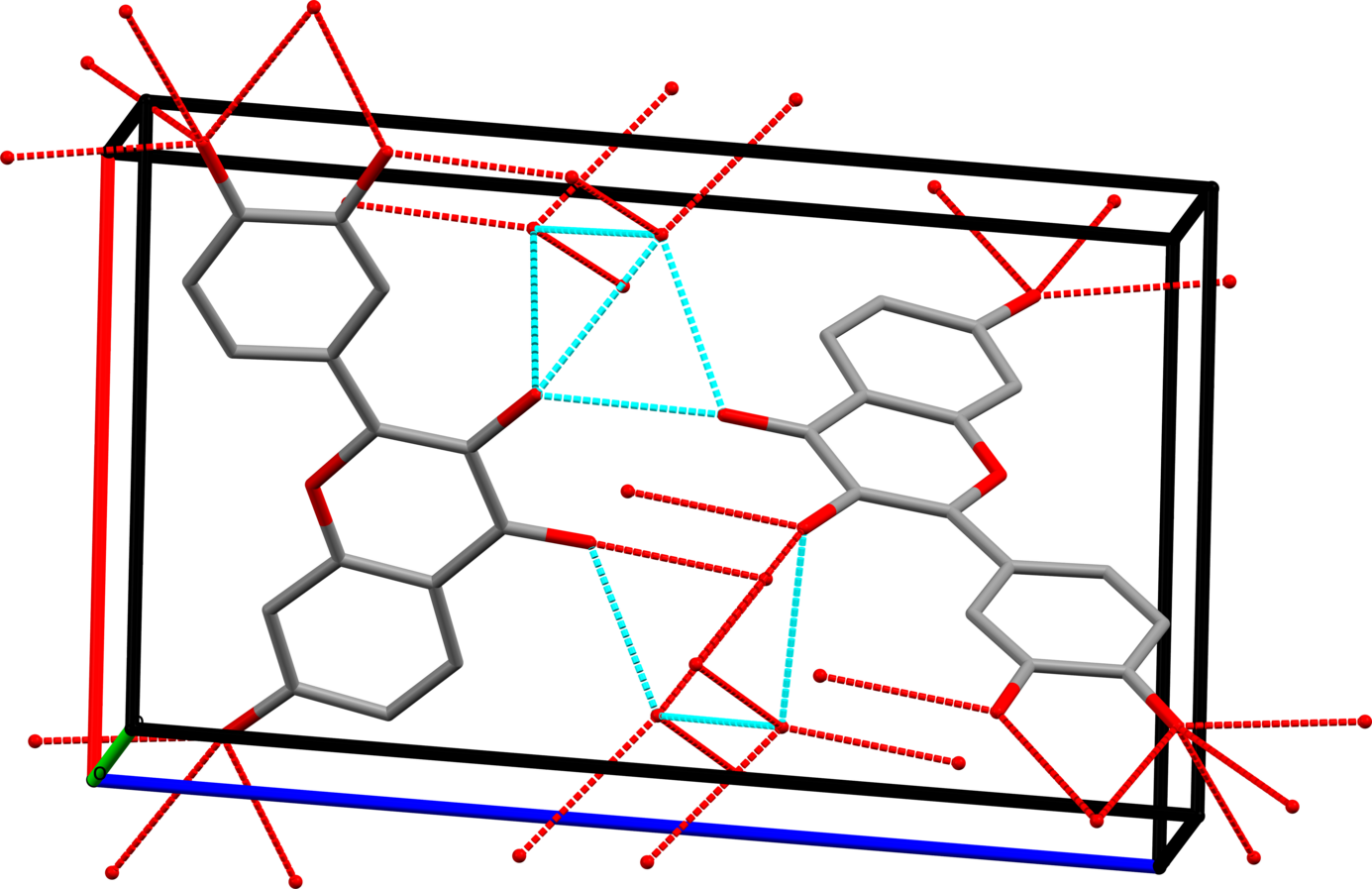
Packing diagram of fisetin dihydrate (**1**) viewed along the *b* axis.

The supra­molecular architecture of the hydrated forms was analyzed based on SCXRD data. Figs. 3 ▸ and 4 ▸ illustrate the unit-cell packing viewed along the *b*-axis direction for both structures.

In fisetin dihydrate (**1**), hydrogen bonding between hydroxyl groups and water mol­ecules generates chains along the *c-*axis direction, which are further linked into layers through additional O—H⋯O inter­actions (Table 1 ▸). In luteolin monohydrate (*2*), water mol­ecules act as hydrogen-bond donors and acceptors, bridging luteolin mol­ecules into an extended three-dimensional network (Table 2 ▸). The disordered water mol­ecule participates in this network through its alternative positions.

**Figure 5:**
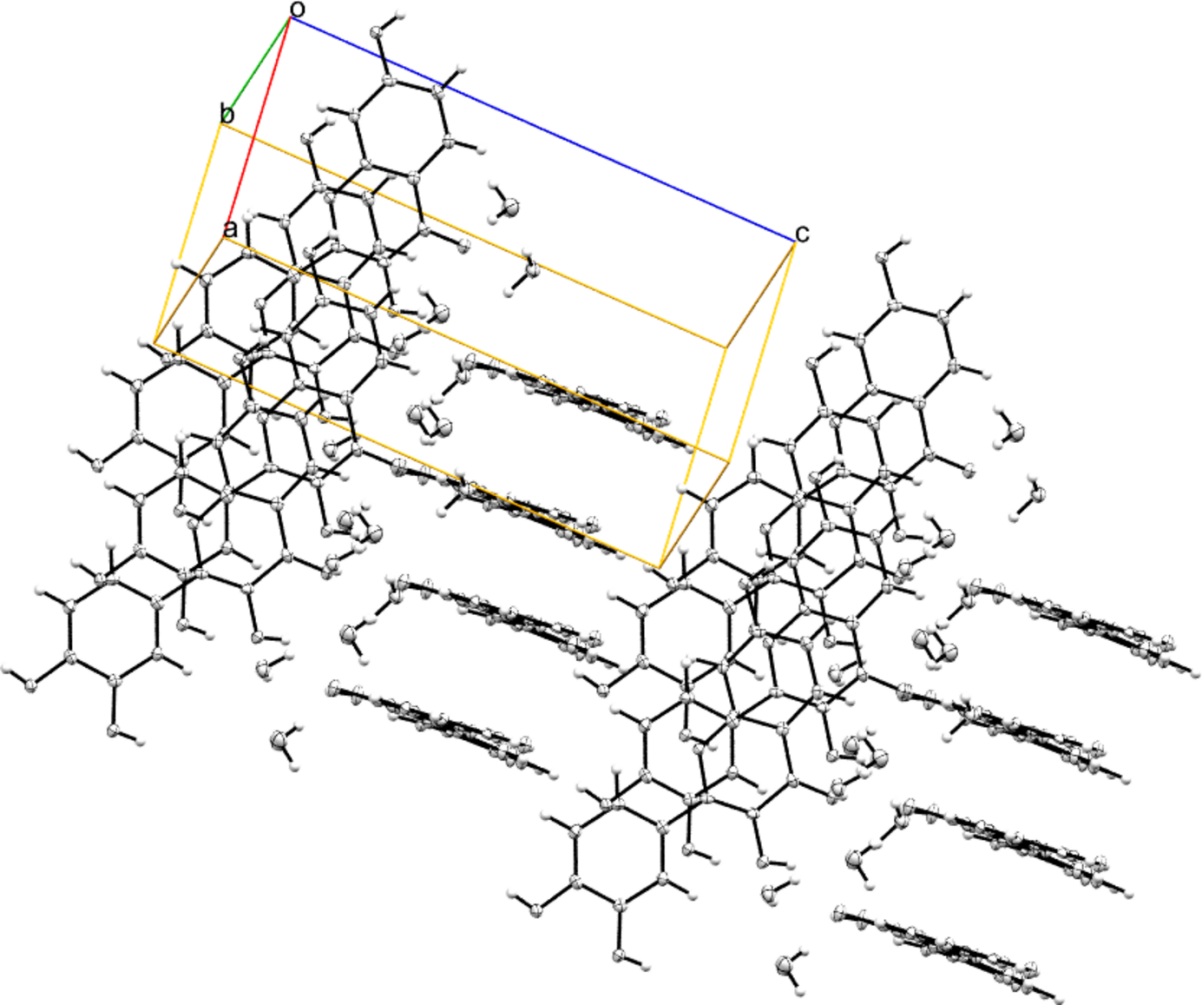
Crystal packing of (**1**), showing the arrangement of the mol­ecules and the relative orientation of the aromatic ring planes involved in π–π stacking inter­actions.

Weak π–π inter­actions are observed in both structures. In (**1**), a short *Cg*1⋯*Cg*2(*x*, *y* + 1, *z*) distance of 3.4210 (15) Å is observed between the O4/C4/C5/C7–C9 and C1–C6 rings (slippage 0.980 Å). In (**2**), the shortest centroid–centroid distance is observed between rings O4/C4/C5/C7–C9 and C10–C15 with *Cg*1⋯*Cg*2(*x* − 1*, y*, *z*) distance of 3.722 (3) Å (slippage 1.715 Å). These contacts are illustrated in Figs. 5 ▸ and 6 ▸. In addition, weak inter­molecular C12—H12⋯*Cg*3(−*x* + 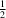, *y* + 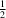, −*z* − 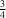) and C7=O3⋯*Cg*3(*x* − 1, *y*, *z)* inter­actions contribute to the consolidation of the crystal packing in (**2**) with a H12⋯*Cg*3 distance of 2.90 Å and O3⋯*Cg*3 separation of 3.428 (4) Å.

## 4. Database survey

The Cambridge Structural Database (CSD version 2025.3; Groom *et al.*, 2016 ▸) contains several entries for fisetin and luteolin. The structures presented in this work are the first reported fisetin dihydrate (**1**) and luteolin monohydrate (**2**).

Studies on related flavonoids, particularly quercetin, have shown that incorporation of water mol­ecules promotes a more planar mol­ecular conformation, facilitating efficient π–π stacking and enhancing crystal stability (Klitou *et al.*, 2019 ▸). A similar trend is observed for fisetin: the anhydrous form (CCDC 1884089; Chadha *et al.*, 2019 ▸) and some co-crystals, such as those with glutaric and malic acid (CCDC 1884086 and 1884087; Cox *et al.*, 2003 ▸), exhibit larger dihedral angles and reduced planarity, whereas others (*e.g.* caffeine, CCDC 986281; Sowa *et al.*, 2014 ▸) are closer to planar; these differences have been linked to variations in stability and water solubility (Chadha *et al.*, 2019 ▸). In contrast, the fisetin dihydrate reported here (**1**) is nearly planar, suggesting a stabilizing effect of hydration.

For luteolin, reported structures [*e.g.* hemihydrate (CCDC 217463; Chadha *et al.*, 2019 ▸) and co-crystals with l/d-proline (CCDC 1444362 and 1446362; He *et al.*, 2016 ▸), and 4,4′-bi­pyridine (CCDC 2385531; Xu *et al.*, 2025 ▸) are predominantly planar, making it difficult to isolate a hydration effect. Nevertheless, they consistently highlight the importance of hydrogen bonding, including water-mediated inter­actions, together with π–π stacking in consolidating the crystal packing.

## 5. Materials and crystallization

**Materials**

Fisetin (3,3′,4′,7-tetra­hydroxy­flavone, CAS 528-48-3, ≥98% purity, Cat. No. CS-7840-25g), luteolin (3′,4′,5,7-tetra­hydroxy­flavone, CAS 491-70-3, ≥98% purity – HPLC, Cat. No. 42437-25g) and 7-hy­droxy­flavone (CAS 6665-86-7, ≥98% purity – Assay, Cat. No. 22027-25g) were purchased from Tzamal D-Chem (Israel). The three compounds are structurally related aglycon flavonoids, and they were subjected to similar ethanol–water crystallization conditions; they produced single crystals of sufficient size and quality for X-ray analysis. The compounds were received as powders and stored under refrigeration (277 K) prior to use.

**Crystal growth**

For each flavonoid, crystals were obtained by preparing a stock solution in absolute ethanol, without additional purification, followed by dilution with double-distilled water (DDW) to reach the desired final ethanol/water ratio. A 4 m*M* stock solution of fisetin in absolute ethanol was diluted with DDW to achieve 20% (*v*/*v*) ethanol/water ratio. The solution, 5 mL total, was incubated in a sealed glass vial at 315 K for 10 days, yielding yellow needle-shaped crystals. A 2 m*M* solution of luteolin in absolute ethanol was diluted with DDW to obtain 2% (*v*/*v*) ethanol/water (5mL total). The solution was placed in an open vial and evaporated at 333 K for 12h, producing colorless needle-shaped crystals. 7-Hy­droxy­flavone monohydrate, a known analogue (Kumar *et al.*, 1998 ▸), was crystallized from a solution of 33% (*v*/*v*) ethanol 4m*M* solution, which was evaporated at 333 K for 12h, yielding colorless needle-shaped crystals.

**Polarized light imaging**

Representative crystals were imaged using an Olympus BX51 optical microscope under cross-polarized light. Samples were prepared by placing the crystals in the original aqueous solution between a glass slide and a cover slip. Images were captured at 10 and 20 × magnifications. Crystal dimensions were measured using *ImageJ* software, version 1.53e (Schneider *et al.*, 2012 ▸).

**Figure 7:**
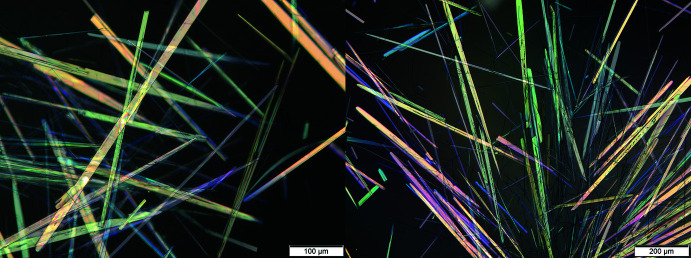
Fisetin dihydrate (**1**) crystals under cross-polarized light.

**Figure 8:**
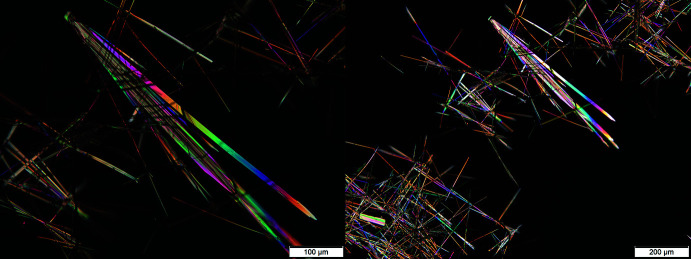
Luteolin monohydrate (**2**) crystals under cross-polarized light.

The polarized light images demonstrate the typical size of the grown crystals. For fisetin dihydrate (**1**) (Fig. 7 ▸), only the larger crystals were measured; the sample also contained smaller particles of approximately 40 µm. Among the larger crystals, lengths ranged from 610–750 µm with thicknesses of 9–18 µm. For luteolin monohydrate (**2**) (Fig. 8 ▸), needle-shaped crystals were observed. Numerous small crystals measured 200–300 µm in length and 2–4 µm in thickness, while a few larger crystals reached 580–600 µm in length and 9–16 µm in thickness.

## 6. Data collection and refinement

A single crystal of yellow needle-shaped C_15_H_14_O_8_ (identified as fisetin dihydrate) (**1**) and a single crystal of colorless block-shaped C_15_H_12_O_7_ (identified as luteolin monohydrate) (*2*) were immersed in Paratone N oil and mounted on a Rigaku Oxford Diffraction XtaLAB Synergy S diffractometer at 100 K. Data collection was carried out using monochromatic Mo *K*_α_ radiation (λ = 0.71073 Å) for (**1**) and Cu *K*_α_ radiation (λ = 1.54184 Å) for (**2**), with φ and ω scans to ensure adequate coverage of reciprocal space. The structures were solved using *Olex2* (Dolomanov *et al.*, 2009 ▸) with the *olex2.solve* algorithm (Bourhis *et al.*, 2015 ▸) (charge flipping) and refined by full-matrix least squares on *F*^2^ using *SHELXL* (Sheldrick, 2015 ▸). All non hydrogen atoms were refined anisotropically. Hydrogen atoms were refined isotopically on calculated positions using a riding model with their *U*_iso_(H) values constrained to 1.5 times the *U*_eq_ of their pivot atoms for terminal *sp*^3^ carbon atoms and 1.2 times for all other carbon atoms. Mol­ecular graphics were prepared using *Mercury* 2022.3.0 (Macrae *et al.*, 2020 ▸). Crystal data, data collection and structure refinement details are summarized in Table 3 ▸.

## Supplementary Material

Crystal structure: contains datablock(s) Neta1R, Neta3R. DOI: 10.1107/S2056989026006353/vm2331sup1.cif

Structure factors: contains datablock(s) Neta1R. DOI: 10.1107/S2056989026006353/vm2331Neta1Rsup2.hkl

Structure factors: contains datablock(s) Neta3R. DOI: 10.1107/S2056989026006353/vm2331Neta3Rsup3.hkl

Supporting information file. DOI: 10.1107/S2056989026006353/vm2331Neta1Rsup4.cml

Supporting information file. DOI: 10.1107/S2056989026006353/vm2331Neta3Rsup5.cml

original CIF file - fisetin. DOI: 10.1107/S2056989026006353/vm2331sup6.txt

original CIF file - luteolin. DOI: 10.1107/S2056989026006353/vm2331sup7.txt

hkl file - fisetin. DOI: 10.1107/S2056989026006353/vm2331sup8.txt

hkl file - luteolin. DOI: 10.1107/S2056989026006353/vm2331sup8.txt

cif file for SCXRD of 7-hydroxyflavone. DOI: 10.1107/S2056989026006353/vm2331sup10.txt

CCDC references: 2484465, 2484464

Additional supporting information:  crystallographic information; 3D view; checkCIF report

## Figures and Tables

**Figure 4:**
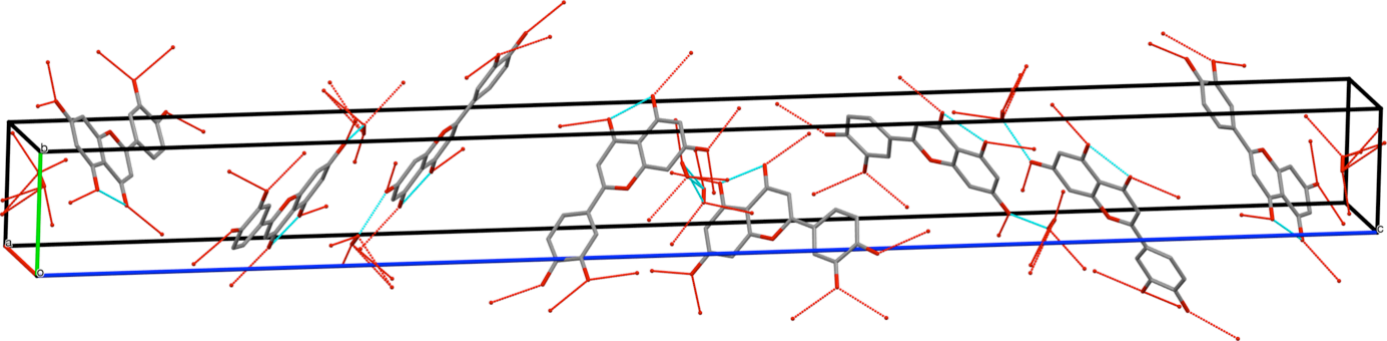
Packing diagram of luteolin monohydrate (**2**) viewed along the *a* axis.

**Figure 6:**
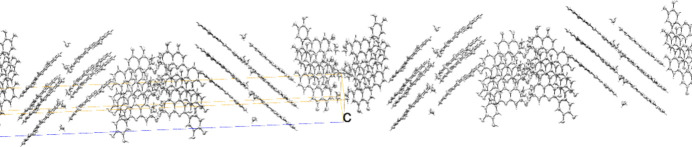
Crystal packing of (**2**), showing the arrangement of the mol­ecules and the relative orientation of the aromatic ring planes involved in π–π stacking inter­actions.

**Table 1:** Hydrogen-bond geometry (Å, °) for Neta1R[Chem scheme1]

*D*—H⋯*A*	*D*—H	H⋯*A*	*D*⋯*A*	*D*—H⋯*A*
O3—H3⋯O2	0.84	2.18	2.638 (3)	114
O3—H3⋯O2^i^	0.84	2.03	2.760 (2)	144
O1—H1⋯O5^ii^	0.84	1.92	2.759 (2)	174
O5—H5⋯O7	0.84	1.86	2.687 (3)	169
O6—H6⋯O1^iii^	0.84	1.96	2.803 (2)	179
O7—H7*A*⋯O8	0.85 (6)	1.82 (6)	2.668 (3)	170 (4)
O7—H7*B*⋯O3^iv^	0.91 (6)	2.32 (6)	3.048 (3)	137 (5)
O7—H7*B*⋯O8^iv^	0.91 (6)	2.27 (6)	3.046 (3)	142 (5)
O8—H8*A*⋯O2^i^	0.91 (5)	1.82 (5)	2.725 (3)	173 (5)
O8—H8*B*⋯O7^v^	0.88 (5)	1.97 (5)	2.831 (3)	167 (6)

Symmetry codes: (i) 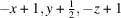; (ii) 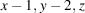; (iii) 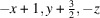; (iv) 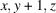; (v) 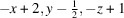.

**Table 2:** Hydrogen-bond geometry (Å, °) for (**2**)

*D*—H⋯*A*	*D*—H	H⋯*A*	*D*⋯*A*	*D*—H⋯*A*
O1—H1⋯O7	0.69 (11)	1.88 (11)	2.571 (17)	174 (14)
O1—H1⋯O7*A*	0.69 (11)	2.43 (11)	3.096 (18)	161 (14)
O2—H2⋯O3	0.96 (10)	1.83 (10)	2.597 (5)	135 (9)
O5—H5⋯O5^i^	0.84	2.50	3.168 (5)	137
O5—H5⋯O6^i^	0.84	2.08	2.824 (5)	147
O6—H6⋯O3^ii^	0.84	1.87	2.694 (5)	165
O7—H7*A*⋯O1^iii^	0.87	2.38	3.112 (19)	142
O7—H7*B*⋯O2^iv^	0.87	1.95	2.811 (15)	173

Symmetry codes: (i) 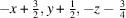; (ii) 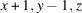; (iii) 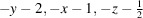; (iv) 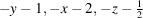.

**Table 3:** Experimental details

	(**1**)	(**2**)/entry>
Crystal data		
Chemical formula	C_15_H_10_O_6_·2H_2_O	C_15_H_10_O_6_·H_2_O
*M*_r_	322.26	304.25
Crystal system, space group	Monoclinic, *P*2_1_	Tetragonal, *P*4_1_2_1_2
Temperature (K)	100	100
*a*, *b*, *c* (Å)	9.0785 (7), 4.7162 (5), 15.3572 (18)	6.1900 (4), 6.1900 (4), 67.344 (6)
α, β, γ (°)	90, 92.369 (9), 90	90, 90, 90
*V* (Å^3^)	656.98 (11)	2580.4 (4)
*Z*	2	8
Radiation type	Mo *K*α	Cu *K*α
μ (mm^−1^)	0.13	1.08
Crystal size (mm)	0.33 × 0.12 × 0.09	0.18 × 0.12 × 0.03

Data collection		
Diffractometer	XtaLAB Synergy-S	XtaLAB Synergy-S
Absorption correction	Multi-scan (*CrysAlis PRO*; Rigaku OD, 2022 ▸)	Multi-scan (*CrysAlis PRO*; Rigaku OD, 2022 ▸)
*T*_min_, *T*_max_	0.937, 0.968	0.809, 0.948
No. of measured, independent and observed [*I* > 2σ(*I*)] reflections	4931, 2638, 2411	5330, 2370, 1881
*R*_int_	0.029	0.063
(sin θ/λ)_max_ (Å^−1^)	0.690	0.614

Refinement		
*R*[*F*^2^ > 2σ(*F*^2^)], *wR*(*F*^2^), *S*	0.034, 0.083, 1.06	0.078, 0.210, 1.01
No. of reflections	2638	2370
No. of parameters	228	225
No. of restraints	1	201
H-atom treatment	H atoms treated by a mixture of independent and constrained refinement	H atoms treated by a mixture of independent and constrained refinement
Δρ_max_, Δρ_min_ (e Å^−3^)	0.23, −0.22	0.33, −0.38
Absolute structure	Flack *x* determined using 853 quotients [(*I*^+^)−(*I*^−^)]/[(*I*^+^)+(*I*^−^)] (Parsons *et al.*, 2013 ▸)	Flack *x* determined using 444 quotients [(*I*^+^)−(*I*^−^)]/[(*I*^+^)+(*I*^−^)] (Parsons *et al.*, 2013 ▸)
Absolute structure parameter	0.03 (7)	0.0 (4)

Computer programs: *CrysAlis PRO* (Rigaku OD, 2022 ▸), *OLEX2.solve* (Bourhis *et al.*, 2015 ▸), *OLEX2* (Dolomanov *et al.*, 2009 ▸), *SHELXL2018/3* (Sheldrick, 2015 ▸) and *Mercury* (Macrae *et al.*, 2020 ▸).

## supplementary crystallographic information

### 2-(3,4-Dihydroxyphenyl)-3,7-dihydroxy-4H-1-benzopyran-4-one dihydrate (Neta1R) Crystal data

**Table:**

C_15_H_10_O_6_·2H_2_O	*F*(000) = 336
*M**_r_* = 322.26	*D*_x_ = 1.629 Mg m^−^^3^
Monoclinic, *P*2_1_	Mo *K*α radiation, λ = 0.71073 Å
*a* = 9.0785 (7) Å	Cell parameters from 2638 reflections
*b* = 4.7162 (5) Å	θ = 2.7–29.4°
*c* = 15.3572 (18) Å	µ = 0.13 mm^−^^1^
β = 92.369 (9)°	*T* = 100 K
*V* = 656.98 (11) Å^3^	Needle, yellow
*Z* = 2	0.33 × 0.12 × 0.09 mm

### 2-(3,4-Dihydroxyphenyl)-3,7-dihydroxy-4H-1-benzopyran-4-one dihydrate (Neta1R) Data collection

**Table:**

XtaLAB Synergy-S diffractometer	2411 reflections with *I* > 2σ(*I*)
Detector resolution: 95 pixels mm^-1^	*R*_int_ = 0.029
ω scans	θ_max_ = 29.4°, θ_min_ = 2.7°
Absorption correction: multi-scan (CrysAlisPro; Rigaku OD, 2022)	*h* = −10→11
*T*_min_ = 0.937, *T*_max_ = 0.968	*k* = −6→5
4931 measured reflections	*l* = −20→18
2638 independent reflections	

### 2-(3,4-Dihydroxyphenyl)-3,7-dihydroxy-4H-1-benzopyran-4-one dihydrate (Neta1R) Refinement

**Table:**

Refinement on *F*^2^	Hydrogen site location: mixed
Least-squares matrix: full	H atoms treated by a mixture of independent and constrained refinement
*R*[*F*^2^ > 2σ(*F*^2^)] = 0.034	*w* = 1/[σ^2^(*F*_o_^2^) + (0.0392*P*)^2^ + 0.1492*P*] where *P* = (*F*_o_^2^ + 2*F*_c_^2^)/3
*wR*(*F*^2^) = 0.083	(Δ/σ)_max_ < 0.001
*S* = 1.06	Δρ_max_ = 0.23 e Å^−^^3^
2638 reflections	Δρ_min_ = −0.21 e Å^−^^3^
228 parameters	Absolute structure: Flack *x* determined using 853 quotients [(*I*^+^)-(*I*^-^)]/[(*I*^+^)+(*I*^-^)] (Parsons *et al.*, 2013)
1 restraint	Absolute structure parameter: 0.03 (7)

### 2-(3,4-Dihydroxyphenyl)-3,7-dihydroxy-4H-1-benzopyran-4-one dihydrate (Neta1R) Special details

**Table:**

Geometry. All esds (except the esd in the dihedral angle between two l.s. planes) are estimated using the full covariance matrix. The cell esds are taken into account individually in the estimation of esds in distances, angles and torsion angles; correlations between esds in cell parameters are only used when they are defined by crystal symmetry. An approximate (isotropic) treatment of cell esds is used for estimating esds involving l.s. planes.

### 2-(3,4-Dihydroxyphenyl)-3,7-dihydroxy-4H-1-benzopyran-4-one dihydrate (Neta1R) Fractional atomic coordinates and isotropic or equivalent isotropic displacement parameters (Å^2^)

**Table:**

	*x*	*y*	*z*	*U*_iso_*/*U*_eq_	
O1	0.1182 (2)	−0.3796 (4)	0.13335 (11)	0.0154 (4)	
H1	0.055562	−0.477165	0.158764	0.023*	
C1	0.1909 (3)	−0.2091 (5)	0.19230 (15)	0.0135 (5)	
O2	0.41137 (19)	0.3567 (4)	0.44683 (10)	0.0173 (4)	
C2	0.1616 (3)	−0.2194 (6)	0.28209 (16)	0.0150 (5)	
H2	0.089780	−0.346481	0.302743	0.018*	
O3	0.6098 (2)	0.6955 (4)	0.38136 (11)	0.0186 (4)	
H3	0.574088	0.690794	0.430930	0.028*	
C3	0.2379 (3)	−0.0443 (6)	0.33871 (15)	0.0148 (5)	
H3A	0.218920	−0.051708	0.399035	0.018*	
O4	0.46911 (18)	0.3350 (4)	0.18641 (10)	0.0124 (4)	
C4	0.3442 (3)	0.1470 (5)	0.30926 (16)	0.0135 (5)	
O5	0.92708 (19)	1.2669 (4)	0.21669 (11)	0.0159 (4)	
H5	0.913091	1.280811	0.270254	0.024*	
C5	0.3700 (3)	0.1531 (5)	0.22029 (16)	0.0117 (5)	
O6	0.9220 (2)	1.2172 (4)	0.04617 (11)	0.0183 (4)	
H6	0.909586	1.191230	−0.007744	0.027*	
C6	0.2953 (3)	−0.0263 (5)	0.16140 (15)	0.0132 (5)	
H6A	0.315788	−0.022870	0.101253	0.016*	
C7	0.4266 (3)	0.3358 (5)	0.36653 (15)	0.0134 (5)	
C8	0.5319 (3)	0.5170 (5)	0.32647 (16)	0.0132 (5)	
C9	0.5501 (3)	0.5200 (5)	0.23852 (15)	0.0120 (5)	
C10	0.6457 (3)	0.7038 (5)	0.18845 (15)	0.0129 (5)	
C11	0.7424 (3)	0.9004 (6)	0.22903 (15)	0.0135 (5)	
H11	0.746773	0.915742	0.290769	0.016*	
C12	0.8315 (3)	1.0725 (5)	0.18035 (15)	0.0127 (5)	
C13	0.8279 (3)	1.0494 (5)	0.08918 (15)	0.0143 (5)	
C14	0.7305 (3)	0.8603 (6)	0.04882 (15)	0.0174 (5)	
H14	0.725433	0.847960	−0.012973	0.021*	
C15	0.6402 (3)	0.6884 (6)	0.09699 (16)	0.0168 (6)	
H15	0.574296	0.559697	0.067981	0.020*	
O7	0.9019 (2)	1.3760 (5)	0.38714 (12)	0.0209 (4)	
H7A	0.879 (5)	1.229 (13)	0.416 (3)	0.070 (16)*	
H7B	0.846 (6)	1.520 (13)	0.408 (4)	0.085 (18)*	
O8	0.8611 (2)	0.9245 (5)	0.48802 (13)	0.0271 (5)	
H8A	0.773 (5)	0.910 (11)	0.514 (3)	0.061 (13)*	
H8B	0.930 (5)	0.934 (13)	0.530 (3)	0.081 (17)*	

### 2-(3,4-Dihydroxyphenyl)-3,7-dihydroxy-4H-1-benzopyran-4-one dihydrate (Neta1R) Atomic displacement parameters (Å^2^)

**Table:**

	*U*^11^	*U*^22^	*U*^33^	*U*^12^	*U*^13^	*U*^23^
O1	0.0184 (10)	0.0155 (10)	0.0124 (8)	−0.0058 (7)	0.0015 (7)	−0.0007 (7)
C1	0.0148 (12)	0.0088 (12)	0.0168 (12)	0.0022 (9)	−0.0006 (9)	0.0000 (9)
O2	0.0202 (9)	0.0200 (10)	0.0117 (8)	−0.0021 (8)	0.0023 (7)	0.0000 (7)
C2	0.0158 (12)	0.0133 (13)	0.0160 (11)	0.0004 (9)	0.0030 (9)	0.0035 (10)
O3	0.0238 (10)	0.0215 (11)	0.0107 (8)	−0.0086 (8)	0.0034 (7)	−0.0036 (8)
C3	0.0178 (13)	0.0148 (13)	0.0120 (11)	0.0024 (10)	0.0034 (9)	0.0016 (10)
O4	0.0155 (8)	0.0114 (9)	0.0104 (7)	−0.0022 (7)	0.0019 (6)	−0.0010 (7)
C4	0.0156 (13)	0.0118 (13)	0.0131 (11)	0.0022 (9)	0.0015 (9)	0.0002 (9)
O5	0.0196 (9)	0.0176 (9)	0.0107 (8)	−0.0043 (7)	0.0018 (6)	−0.0014 (7)
C5	0.0115 (11)	0.0091 (13)	0.0147 (11)	0.0009 (9)	0.0010 (9)	0.0019 (9)
O6	0.0241 (10)	0.0187 (10)	0.0123 (8)	−0.0082 (8)	0.0044 (7)	−0.0010 (8)
C6	0.0169 (12)	0.0119 (12)	0.0108 (11)	0.0025 (9)	0.0021 (9)	0.0014 (9)
C7	0.0130 (11)	0.0120 (13)	0.0153 (11)	0.0027 (10)	0.0028 (9)	0.0034 (10)
C8	0.0146 (12)	0.0103 (13)	0.0148 (11)	−0.0009 (9)	0.0001 (9)	−0.0010 (9)
C9	0.0115 (11)	0.0091 (12)	0.0152 (11)	0.0008 (9)	0.0001 (9)	−0.0016 (9)
C10	0.0125 (12)	0.0103 (12)	0.0161 (11)	0.0022 (9)	0.0036 (9)	0.0010 (10)
C11	0.0140 (11)	0.0146 (13)	0.0120 (10)	0.0016 (10)	0.0021 (9)	−0.0001 (9)
C12	0.0133 (12)	0.0101 (12)	0.0147 (11)	0.0021 (9)	0.0004 (9)	−0.0009 (10)
C13	0.0156 (13)	0.0122 (13)	0.0154 (11)	−0.0004 (10)	0.0052 (9)	0.0024 (10)
C14	0.0225 (13)	0.0186 (14)	0.0111 (10)	−0.0019 (11)	0.0030 (9)	−0.0020 (10)
C15	0.0188 (13)	0.0157 (14)	0.0158 (12)	−0.0051 (10)	0.0006 (10)	−0.0022 (10)
O7	0.0255 (10)	0.0210 (11)	0.0166 (9)	0.0010 (9)	0.0046 (7)	−0.0001 (8)
O8	0.0216 (11)	0.0365 (13)	0.0236 (10)	−0.0001 (9)	0.0042 (8)	0.0040 (9)

### 2-(3,4-Dihydroxyphenyl)-3,7-dihydroxy-4H-1-benzopyran-4-one dihydrate (Neta1R) Geometric parameters (Å, º)

**Table:**

O1—C1	1.361 (3)	O6—H6	0.8400
O1—H1	0.8400	C6—H6A	0.9500
C1—C6	1.379 (3)	C7—C8	1.439 (3)
C1—C2	1.416 (3)	C8—C9	1.367 (3)
O2—C7	1.251 (3)	C9—C10	1.467 (3)
C2—C3	1.367 (4)	C10—C11	1.405 (4)
C2—H2	0.9500	C10—C15	1.405 (3)
O3—C8	1.367 (3)	C11—C12	1.386 (3)
O3—H3	0.8400	C11—H11	0.9500
C3—C4	1.409 (4)	C12—C13	1.403 (3)
C3—H3A	0.9500	C13—C14	1.384 (4)
O4—C5	1.362 (3)	C14—C15	1.388 (4)
O4—C9	1.376 (3)	C14—H14	0.9500
C4—C5	1.396 (3)	C15—H15	0.9500
C4—C7	1.439 (4)	O7—H7A	0.86 (6)
O5—C12	1.366 (3)	O7—H7B	0.91 (6)
O5—H5	0.8400	O8—H8A	0.91 (5)
C5—C6	1.394 (3)	O8—H8B	0.88 (5)
O6—C13	1.356 (3)		
			
C1—O1—H1	109.5	O3—C8—C9	121.5 (2)
O1—C1—C6	117.5 (2)	O3—C8—C7	116.0 (2)
O1—C1—C2	121.5 (2)	C9—C8—C7	122.5 (2)
C6—C1—C2	121.0 (2)	C8—C9—O4	119.0 (2)
C3—C2—C1	119.2 (2)	C8—C9—C10	128.5 (2)
C3—C2—H2	120.4	O4—C9—C10	112.55 (19)
C1—C2—H2	120.4	C11—C10—C15	118.3 (2)
C8—O3—H3	109.5	C11—C10—C9	122.0 (2)
C2—C3—C4	121.2 (2)	C15—C10—C9	119.7 (2)
C2—C3—H3A	119.4	C12—C11—C10	121.0 (2)
C4—C3—H3A	119.4	C12—C11—H11	119.5
C5—O4—C9	121.52 (18)	C10—C11—H11	119.5
C5—C4—C3	118.2 (2)	O5—C12—C11	123.2 (2)
C5—C4—C7	118.8 (2)	O5—C12—C13	116.6 (2)
C3—C4—C7	123.1 (2)	C11—C12—C13	120.1 (2)
C12—O5—H5	109.5	O6—C13—C14	124.2 (2)
O4—C5—C6	116.5 (2)	O6—C13—C12	116.8 (2)
O4—C5—C4	121.8 (2)	C14—C13—C12	119.0 (2)
C6—C5—C4	121.7 (2)	C13—C14—C15	121.2 (2)
C13—O6—H6	109.5	C13—C14—H14	119.4
C1—C6—C5	118.6 (2)	C15—C14—H14	119.4
C1—C6—H6A	120.7	C14—C15—C10	120.3 (2)
C5—C6—H6A	120.7	C14—C15—H15	119.8
O2—C7—C8	118.5 (2)	C10—C15—H15	119.8
O2—C7—C4	125.1 (2)	H7A—O7—H7B	106 (4)
C8—C7—C4	116.4 (2)	H8A—O8—H8B	107 (4)
			
O1—C1—C2—C3	179.8 (2)	O3—C8—C9—O4	179.8 (2)
C6—C1—C2—C3	0.2 (4)	C7—C8—C9—O4	−2.7 (4)
C1—C2—C3—C4	0.4 (4)	O3—C8—C9—C10	−1.6 (4)
C2—C3—C4—C5	−0.1 (4)	C7—C8—C9—C10	175.9 (2)
C2—C3—C4—C7	179.7 (2)	C5—O4—C9—C8	1.1 (3)
C9—O4—C5—C6	−179.4 (2)	C5—O4—C9—C10	−177.8 (2)
C9—O4—C5—C4	0.7 (3)	C8—C9—C10—C11	4.0 (4)
C3—C4—C5—O4	178.9 (2)	O4—C9—C10—C11	−177.3 (2)
C7—C4—C5—O4	−0.9 (3)	C8—C9—C10—C15	−175.0 (3)
C3—C4—C5—C6	−0.9 (4)	O4—C9—C10—C15	3.7 (3)
C7—C4—C5—C6	179.2 (2)	C15—C10—C11—C12	−0.8 (4)
O1—C1—C6—C5	179.2 (2)	C9—C10—C11—C12	−179.9 (2)
C2—C1—C6—C5	−1.2 (4)	C10—C11—C12—O5	179.9 (2)
O4—C5—C6—C1	−178.3 (2)	C10—C11—C12—C13	−0.9 (4)
C4—C5—C6—C1	1.6 (4)	O5—C12—C13—O6	1.6 (3)
C5—C4—C7—O2	178.0 (2)	C11—C12—C13—O6	−177.6 (2)
C3—C4—C7—O2	−1.8 (4)	O5—C12—C13—C14	−178.5 (2)
C5—C4—C7—C8	−0.6 (3)	C11—C12—C13—C14	2.3 (4)
C3—C4—C7—C8	179.6 (2)	O6—C13—C14—C15	178.0 (2)
O2—C7—C8—O3	1.4 (3)	C12—C13—C14—C15	−1.9 (4)
C4—C7—C8—O3	−179.9 (2)	C13—C14—C15—C10	0.2 (4)
O2—C7—C8—C9	−176.3 (2)	C11—C10—C15—C14	1.2 (4)
C4—C7—C8—C9	2.4 (4)	C9—C10—C15—C14	−179.7 (2)

### 2-(3,4-Dihydroxyphenyl)-3,7-dihydroxy-4H-1-benzopyran-4-one dihydrate (Neta1R) Hydrogen-bond geometry (Å, º)

**Table:**

*D*—H···*A*	*D*—H	H···*A*	*D*···*A*	*D*—H···*A*
O3—H3···O2	0.84	2.18	2.638 (3)	114
O3—H3···O2^i^	0.84	2.03	2.760 (2)	144
O1—H1···O5^ii^	0.84	1.92	2.759 (2)	174
O5—H5···O7	0.84	1.86	2.687 (3)	169
O6—H6···O1^iii^	0.84	1.96	2.803 (2)	179
O7—H7*A*···O8	0.85 (6)	1.82 (6)	2.668 (3)	170 (4)
O7—H7*B*···O3^iv^	0.91 (6)	2.32 (6)	3.048 (3)	137 (5)
O7—H7*B*···O8^iv^	0.91 (6)	2.27 (6)	3.046 (3)	142 (5)
O8—H8*A*···O2^i^	0.91 (5)	1.82 (5)	2.725 (3)	173 (5)
O8—H8*B*···O7^v^	0.88 (5)	1.97 (5)	2.831 (3)	167 (6)

Symmetry codes: (i) −*x*+1, *y*+1/2, −*z*+1; (ii) *x*−1, *y*−2, *z*; (iii) −*x*+1, *y*+3/2, −*z*; (iv) *x*, *y*+1, *z*; (v) −*x*+2, *y*−1/2, −*z*+1.

### 2-(3,4-Dihydroxyphenyl)-5,7-dihydroxy-4H-chromen-4-one monohydrate (Neta3R) Crystal data

**Table:**

C_15_H_10_O_6_·H_2_O	*D*_x_ = 1.566 Mg m^−^^3^
*M**_r_* = 304.25	Cu *K*α radiation, λ = 1.54184 Å
Tetragonal, *P*4_1_2_1_2	Cell parameters from 2370 reflections
*a* = 6.1900 (4) Å	θ = 2.6–71.3°
*c* = 67.344 (6) Å	µ = 1.08 mm^−^^1^
*V* = 2580.4 (4) Å^3^	*T* = 100 K
*Z* = 8	Plate, light yellow
*F*(000) = 1264	0.18 × 0.12 × 0.03 mm

### 2-(3,4-Dihydroxyphenyl)-5,7-dihydroxy-4H-chromen-4-one monohydrate (Neta3R) Data collection

**Table:**

XtaLAB Synergy-S diffractometer	1881 reflections with *I* > 2σ(*I*)
Detector resolution: 95 pixels mm^-1^	*R*_int_ = 0.063
ω scans	θ_max_ = 71.3°, θ_min_ = 2.6°
Absorption correction: multi-scan (CrysAlisPro; Rigaku OD, 2022)	*h* = −7→7
*T*_min_ = 0.809, *T*_max_ = 0.948	*k* = −7→3
5330 measured reflections	*l* = −81→77
2370 independent reflections	

### 2-(3,4-Dihydroxyphenyl)-5,7-dihydroxy-4H-chromen-4-one monohydrate (Neta3R) Refinement

**Table:**

Refinement on *F*^2^	Hydrogen site location: mixed
Least-squares matrix: full	H atoms treated by a mixture of independent and constrained refinement
*R*[*F*^2^ > 2σ(*F*^2^)] = 0.078	*w* = 1/[σ^2^(*F*_o_^2^) + (0.1556*P*)^2^] where *P* = (*F*_o_^2^ + 2*F*_c_^2^)/3
*wR*(*F*^2^) = 0.210	(Δ/σ)_max_ < 0.001
*S* = 1.01	Δρ_max_ = 0.33 e Å^−^^3^
2370 reflections	Δρ_min_ = −0.38 e Å^−^^3^
225 parameters	Absolute structure: Flack *x* determined using 444 quotients [(*I*^+^)-(*I*^-^)]/[(*I*^+^)+(*I*^-^)] (Parsons *et al.*, 2013)
201 restraints	Absolute structure parameter: 0.0 (4)

### 2-(3,4-Dihydroxyphenyl)-5,7-dihydroxy-4H-chromen-4-one monohydrate (Neta3R) Special details

**Table:**

Geometry. All esds (except the esd in the dihedral angle between two l.s. planes) are estimated using the full covariance matrix. The cell esds are taken into account individually in the estimation of esds in distances, angles and torsion angles; correlations between esds in cell parameters are only used when they are defined by crystal symmetry. An approximate (isotropic) treatment of cell esds is used for estimating esds involving l.s. planes.

### 2-(3,4-Dihydroxyphenyl)-5,7-dihydroxy-4H-chromen-4-one monohydrate (Neta3R) Fractional atomic coordinates and isotropic or equivalent isotropic displacement parameters (Å^2^)

**Table:**

	*x*	*y*	*z*	*U*_iso_*/*U*_eq_	Occ. (<1)
O1	−0.4385 (10)	−0.8908 (9)	−0.26178 (7)	0.0725 (16)	
H1	−0.532 (18)	−0.91 (2)	−0.2564 (15)	0.13 (4)*	
C1	−0.4259 (10)	−0.7179 (10)	−0.27429 (7)	0.0485 (14)	
O2	−0.7251 (6)	−0.2359 (8)	−0.28783 (5)	0.0541 (11)	
H2	−0.707 (17)	−0.118 (16)	−0.2969 (14)	0.15 (4)*	
C2	−0.5853 (9)	−0.5633 (10)	−0.27473 (7)	0.0492 (13)	
H2A	−0.707076	−0.576830	−0.266238	0.059*	
O3	−0.5013 (5)	−0.0446 (6)	−0.31530 (5)	0.0435 (9)	
C3	−0.5700 (8)	−0.3884 (10)	−0.28743 (6)	0.0435 (12)	
O4	−0.0589 (5)	−0.5338 (6)	−0.31209 (4)	0.0390 (9)	
C4	−0.3894 (8)	−0.3711 (9)	−0.30033 (6)	0.0366 (11)	
O5	0.7062 (5)	−0.4891 (6)	−0.37203 (5)	0.0415 (9)	
H5	0.726959	−0.386452	−0.379962	0.062*	
C5	−0.2368 (8)	−0.5324 (9)	−0.29957 (6)	0.0392 (11)	
O6	0.6085 (6)	−0.7754 (6)	−0.34507 (5)	0.0422 (9)	
H6	0.582565	−0.841522	−0.334441	0.063*	
C6	−0.2453 (9)	−0.7091 (9)	−0.28681 (7)	0.0471 (13)	
H6A	−0.135908	−0.816755	−0.286611	0.057*	
C7	−0.3644 (8)	−0.1943 (9)	−0.31414 (6)	0.0369 (11)	
C8	−0.1746 (7)	−0.2060 (9)	−0.32620 (7)	0.0362 (11)	
H8	−0.146813	−0.092164	−0.335309	0.043*	
C9	−0.0355 (8)	−0.3691 (8)	−0.32521 (6)	0.0343 (10)	
C10	0.1585 (7)	−0.3974 (8)	−0.33758 (6)	0.0326 (10)	
C11	0.2118 (8)	−0.2461 (9)	−0.35198 (7)	0.0403 (11)	
H11	0.122048	−0.123224	−0.353848	0.048*	
C12	0.3928 (8)	−0.2717 (9)	−0.36356 (7)	0.0393 (12)	
H12	0.427458	−0.166304	−0.373304	0.047*	
C13	0.5246 (8)	−0.4497 (8)	−0.36113 (6)	0.0357 (10)	
C14	0.4725 (7)	−0.6052 (7)	−0.34678 (6)	0.0322 (10)	
C15	0.2900 (7)	−0.5792 (8)	−0.33526 (6)	0.0333 (10)	
H15	0.253465	−0.685700	−0.325668	0.040*	
O7A	−0.865 (3)	−0.848 (4)	−0.23808 (18)	0.035 (5)	0.32 (4)
H7AA	−0.949950	−0.908568	−0.246730	0.053*	0.32 (4)
H7AB	−0.903404	−0.712675	−0.238077	0.053*	0.32 (4)
O7	−0.778 (3)	−0.939 (3)	−0.24023 (11)	0.066 (4)	0.68 (4)
H7A	−0.840646	−0.831929	−0.234122	0.100*	0.68 (4)
H7B	−0.764025	−1.038062	−0.231160	0.100*	0.68 (4)

### 2-(3,4-Dihydroxyphenyl)-5,7-dihydroxy-4H-chromen-4-one monohydrate (Neta3R) Atomic displacement parameters (Å^2^)

**Table:**

	*U*^11^	*U*^22^	*U*^33^	*U*^12^	*U*^13^	*U*^23^
O1	0.100 (4)	0.067 (3)	0.051 (2)	−0.045 (3)	0.019 (2)	0.009 (2)
C1	0.052 (3)	0.054 (3)	0.040 (3)	−0.025 (3)	0.007 (2)	−0.003 (2)
O2	0.035 (2)	0.078 (3)	0.050 (2)	−0.011 (2)	0.0090 (16)	−0.0023 (19)
C2	0.040 (3)	0.071 (4)	0.037 (2)	−0.030 (3)	0.011 (2)	−0.003 (2)
O3	0.0324 (19)	0.047 (2)	0.0514 (19)	−0.0091 (17)	0.0068 (14)	−0.0030 (15)
C3	0.027 (3)	0.064 (4)	0.039 (2)	−0.019 (2)	0.0033 (18)	−0.008 (2)
O4	0.0365 (19)	0.043 (2)	0.0376 (16)	−0.0103 (16)	0.0050 (13)	0.0040 (13)
C4	0.029 (2)	0.049 (3)	0.031 (2)	−0.019 (2)	0.0008 (17)	−0.0026 (18)
O5	0.033 (2)	0.047 (2)	0.0444 (18)	−0.0095 (17)	0.0100 (13)	0.0015 (15)
C5	0.039 (3)	0.046 (3)	0.033 (2)	−0.018 (2)	0.0045 (18)	−0.0052 (18)
O6	0.0370 (19)	0.037 (2)	0.0527 (19)	−0.0037 (16)	0.0140 (15)	0.0001 (14)
C6	0.053 (3)	0.049 (3)	0.039 (2)	−0.021 (3)	0.008 (2)	−0.001 (2)
C7	0.025 (2)	0.049 (3)	0.037 (2)	−0.010 (2)	−0.0004 (17)	−0.004 (2)
C8	0.021 (2)	0.049 (3)	0.038 (2)	−0.010 (2)	0.0018 (17)	0.0065 (19)
C9	0.027 (2)	0.043 (3)	0.033 (2)	−0.012 (2)	−0.0003 (17)	0.0005 (18)
C10	0.025 (2)	0.039 (3)	0.034 (2)	−0.012 (2)	−0.0004 (16)	0.0014 (17)
C11	0.030 (2)	0.052 (3)	0.039 (2)	0.002 (2)	−0.0010 (18)	0.011 (2)
C12	0.026 (2)	0.053 (3)	0.039 (2)	−0.009 (2)	0.0001 (18)	0.007 (2)
C13	0.028 (2)	0.044 (3)	0.035 (2)	−0.011 (2)	0.0011 (16)	−0.0041 (18)
C14	0.026 (2)	0.031 (2)	0.039 (2)	−0.0047 (19)	0.0018 (17)	−0.0023 (17)
C15	0.025 (2)	0.039 (3)	0.037 (2)	−0.011 (2)	0.0002 (16)	−0.0020 (17)
O7A	0.034 (8)	0.029 (8)	0.042 (6)	0.007 (7)	0.012 (5)	0.002 (5)
O7	0.081 (8)	0.057 (7)	0.061 (4)	0.002 (7)	0.014 (4)	0.007 (4)

### 2-(3,4-Dihydroxyphenyl)-5,7-dihydroxy-4H-chromen-4-one monohydrate (Neta3R) Geometric parameters (Å, º)

**Table:**

O1—C1	1.365 (7)	C6—H6A	0.9500
O1—H1	0.69 (10)	C7—C8	1.429 (6)
C1—C2	1.375 (8)	C8—C9	1.328 (7)
C1—C6	1.402 (7)	C8—H8	0.9500
O2—C3	1.347 (7)	C9—C10	1.472 (6)
O2—H2	0.96 (10)	C10—C11	1.388 (6)
C2—C3	1.383 (8)	C10—C15	1.397 (7)
C2—H2A	0.9500	C11—C12	1.375 (7)
O3—C7	1.258 (6)	C11—H11	0.9500
C3—C4	1.420 (6)	C12—C13	1.381 (7)
O4—C9	1.357 (6)	C12—H12	0.9500
O4—C5	1.387 (6)	C13—C14	1.401 (6)
C4—C5	1.376 (7)	C14—C15	1.380 (6)
C4—C7	1.445 (7)	C15—H15	0.9500
O5—C13	1.364 (6)	O7A—H7AA	0.8700
O5—H5	0.8400	O7A—H7AB	0.8700
C5—C6	1.392 (7)	O7—H7A	0.8700
O6—C14	1.354 (6)	O7—H7B	0.8701
O6—H6	0.8400		
			
C1—O1—H1	120 (10)	C9—C8—C7	122.8 (5)
O1—C1—C2	121.2 (5)	C9—C8—H8	118.6
O1—C1—C6	116.6 (6)	C7—C8—H8	118.6
C2—C1—C6	122.2 (5)	C8—C9—O4	122.3 (4)
C3—O2—H2	118 (7)	C8—C9—C10	126.2 (5)
C1—C2—C3	120.6 (5)	O4—C9—C10	111.4 (4)
C1—C2—H2A	119.7	C11—C10—C15	118.9 (4)
C3—C2—H2A	119.7	C11—C10—C9	120.6 (5)
O2—C3—C2	120.8 (5)	C15—C10—C9	120.5 (4)
O2—C3—C4	119.8 (5)	C12—C11—C10	120.8 (5)
C2—C3—C4	119.4 (6)	C12—C11—H11	119.6
C9—O4—C5	118.4 (4)	C10—C11—H11	119.6
C5—C4—C3	117.6 (5)	C11—C12—C13	120.5 (5)
C5—C4—C7	120.0 (4)	C11—C12—H12	119.8
C3—C4—C7	122.4 (5)	C13—C12—H12	119.8
C13—O5—H5	109.5	O5—C13—C12	124.4 (4)
C4—C5—O4	121.8 (4)	O5—C13—C14	116.0 (4)
C4—C5—C6	124.6 (5)	C12—C13—C14	119.6 (4)
O4—C5—C6	113.6 (5)	O6—C14—C15	123.5 (4)
C14—O6—H6	109.5	O6—C14—C13	116.8 (4)
C5—C6—C1	115.6 (6)	C15—C14—C13	119.8 (4)
C5—C6—H6A	122.2	C14—C15—C10	120.5 (4)
C1—C6—H6A	122.2	C14—C15—H15	119.8
O3—C7—C8	123.7 (5)	C10—C15—H15	119.8
O3—C7—C4	121.7 (4)	H7AA—O7A—H7AB	104.5
C8—C7—C4	114.6 (5)	H7A—O7—H7B	104.5
			
O1—C1—C2—C3	179.7 (5)	C4—C7—C8—C9	1.8 (7)
C6—C1—C2—C3	−1.6 (8)	C7—C8—C9—O4	−2.5 (7)
C1—C2—C3—O2	−179.6 (4)	C7—C8—C9—C10	177.5 (4)
C1—C2—C3—C4	1.1 (8)	C5—O4—C9—C8	1.2 (6)
O2—C3—C4—C5	−179.1 (4)	C5—O4—C9—C10	−178.9 (3)
C2—C3—C4—C5	0.3 (7)	C8—C9—C10—C11	0.0 (7)
O2—C3—C4—C7	−0.2 (7)	O4—C9—C10—C11	−180.0 (4)
C2—C3—C4—C7	179.2 (4)	C8—C9—C10—C15	−178.9 (4)
C3—C4—C5—O4	177.6 (4)	O4—C9—C10—C15	1.2 (6)
C7—C4—C5—O4	−1.4 (7)	C15—C10—C11—C12	−1.1 (7)
C3—C4—C5—C6	−1.2 (7)	C9—C10—C11—C12	180.0 (4)
C7—C4—C5—C6	179.9 (4)	C10—C11—C12—C13	0.3 (8)
C9—O4—C5—C4	0.8 (6)	C11—C12—C13—O5	179.5 (4)
C9—O4—C5—C6	179.7 (4)	C11—C12—C13—C14	0.2 (7)
C4—C5—C6—C1	0.7 (7)	O5—C13—C14—O6	0.9 (6)
O4—C5—C6—C1	−178.2 (4)	C12—C13—C14—O6	−179.8 (4)
O1—C1—C6—C5	179.4 (5)	O5—C13—C14—C15	−179.2 (4)
C2—C1—C6—C5	0.7 (8)	C12—C13—C14—C15	0.1 (7)
C5—C4—C7—O3	179.7 (4)	O6—C14—C15—C10	178.9 (4)
C3—C4—C7—O3	0.8 (7)	C13—C14—C15—C10	−0.9 (6)
C5—C4—C7—C8	0.1 (6)	C11—C10—C15—C14	1.5 (6)
C3—C4—C7—C8	−178.8 (4)	C9—C10—C15—C14	−179.7 (4)
O3—C7—C8—C9	−177.8 (4)		

### 2-(3,4-Dihydroxyphenyl)-5,7-dihydroxy-4H-chromen-4-one monohydrate (Neta3R) Hydrogen-bond geometry (Å, º)

**Table:**

*D*—H···*A*	*D*—H	H···*A*	*D*···*A*	*D*—H···*A*
O1—H1···O7	0.69 (11)	1.88 (11)	2.571 (17)	174 (14)
O1—H1···O7*A*	0.69 (11)	2.43 (11)	3.096 (18)	161 (14)
O2—H2···O3	0.96 (10)	1.83 (10)	2.597 (5)	135 (9)
O5—H5···O5^i^	0.84	2.50	3.168 (5)	137
O5—H5···O6^i^	0.84	2.08	2.824 (5)	147
O6—H6···O3^ii^	0.84	1.87	2.694 (5)	165
O7—H7*A*···O1^iii^	0.87	2.38	3.112 (19)	142
O7—H7*B*···O2^iv^	0.87	1.95	2.811 (15)	173

Symmetry codes: (i) −*x*+3/2, *y*+1/2, −*z*−3/4; (ii) *x*+1, *y*−1, *z*; (iii) −*y*−2, −*x*−1, −*z*−1/2; (iv) −*y*−1, −*x*−2, −*z*−1/2.
